# Supplementary material for: The proteomic landscape of stool-derived extracellular vesicles in patients with pre-cancerous lesions and colorectal cancer
Source: Commun Biol. 2025 Feb 13;8:228. doi: 10.1038/s42003-025-07652-5 (PMC11825688; doi:10.1038/s42003-025-07652-5)
Supplement: Supplementary file 1 — Supplementary Material [file 42003_2025_7652_MOESM1_ESM.pdf]

Supplementary Table 1.

Requirement	Please Include Requested Information
1.1. Purpose	Determine particle recovery following size-exclusion chromatography and ultrafiltration of stool EV preparations
1.2. Keywords	Size exclusion chromatography, Ultrafiltration
1.3. Experiment variables	SEC pore size and UF input volumes
1.4. Organization name and address	Mayo Clinic<br>200 1st Street SW<br>Rochester, MN 55905
1.5. Primary contact name and email address	Fabrice Lucien Lucien-Matteoni.Fabrice@mayo.edu
1.6. Date or time period of experiment	September-October 2022
1.7. Conclusions	1) There was no difference in SEC particle recovery among Izon's 70nm and 35nm columns<br>2) UF-15ml centrifugal filters had increased recovery compared to UF-4ml filters
1.8. Quality control measures	Side-scatter (LALS) intensities in arbitrary unit were converted to standardized unit in nm with the use of Rosetta Calibration (#Cal003, Exometry, Amsterdam, The Netherlands).
1.9 Other relevant experiment information	
2.1 Sample description	Pooled SEC fractions, UF concentrates
2.1.1 Biological sample source description	EVs derived from stool supernatant
2.1.2 Biological sample source organism description	Human (n=4)
2.2 Sample characteristics	Cancer-free patients
2.3. Sample treatment description	Samples were diluted in PBS (1:1000-1:2000)
2.4. Fluorescence reagent(s) description	NA
3.1. Instrument manufacturer	Apogee, Northwood, UK
3.2. Instrument model	A60-Micro Plus
3.3. Instrument configuration and settings	Samples were analyzed at a flow rate of 0.75 µl/min on an A60-Micro Plus, equipped with a 405 nm laser (70 mW), 488 nm laser (70 mW), 647 nm laser (70 mW). Samples were measured for 1 minute with 405-nm side scatter (LALS) using triggering thresholds of 1700 a.u.
3.4 Instrument light scatter detection limits	We used triggering threshold on side scatter (LALS) only because of the higher sensitivity for small particles. It was set at 1700 arbitrary units which corresponds to a scattering cross section of 12 nm^2 and an EV diameter of 168 nm, given the assumed refractive index distribution of an EV by Mie theory modeling (Line). The upper limit is 4.1 x 10^6 arbitrary units corresponds to a scattering cross section of 40,000 nm^2 and an EV diameter of 4600 nm.
4.1. List-mode data files	
4.2. Compensation description	
4.3. Data transformation details	FlowJo (v10.6.2; FlowJo, Ashland, OR) and Rosetta Calibration software (Purchased license, Exometry, Amsterdam, The Netherlands) were used to transform Data acquired from Apogee Micro 60 Plus.

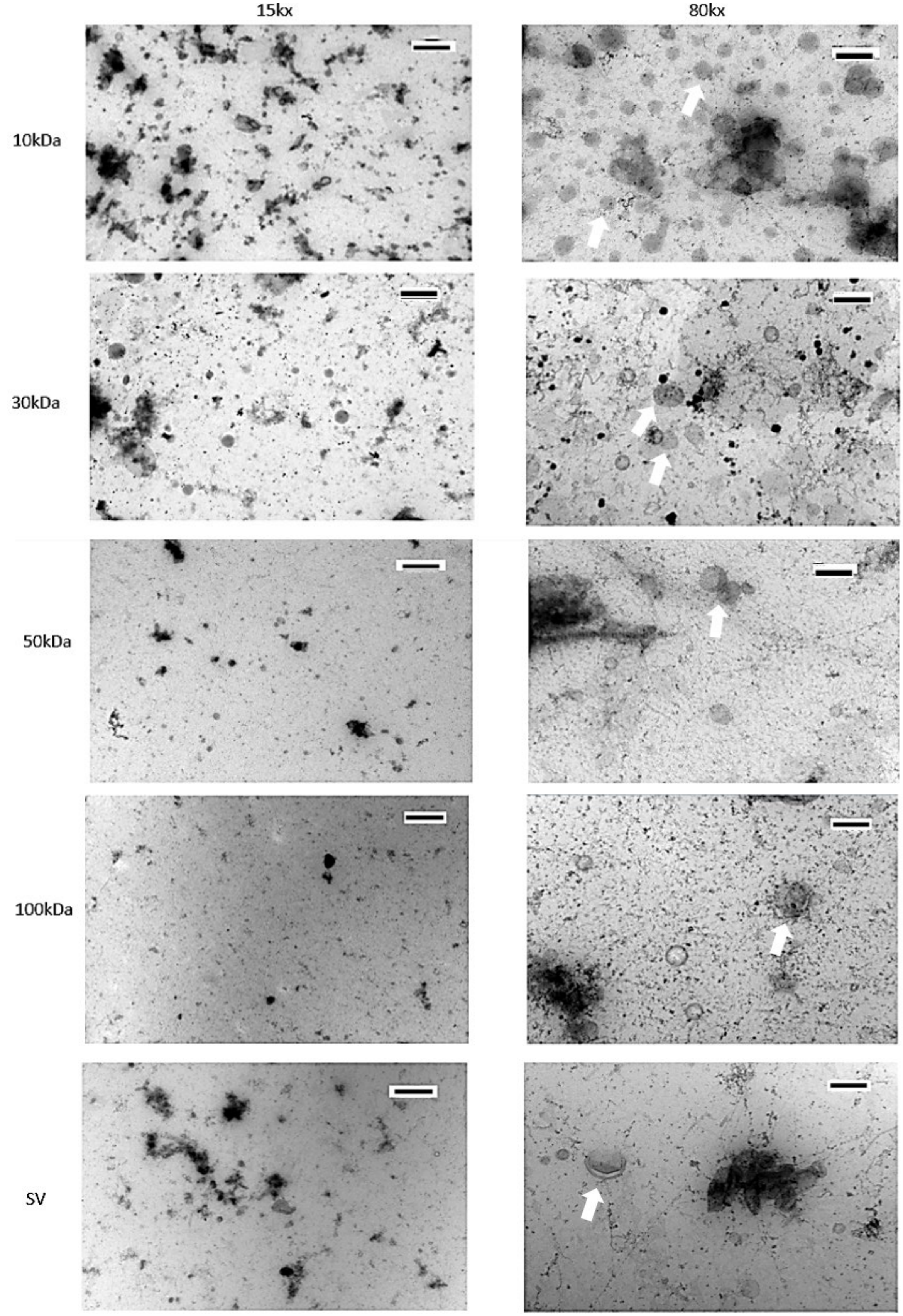

Supplementary Figure 1. Representative EM pictures showing EVs that were isolated by SEC and further concentrated by ultrafiltration at various MWCOs or speed vacuum captured separately at 15kx and 80kx. Lower magnification shows a wider field of view and a better representation of contaminants in samples. White arrows at higher magnification indicate individual EVs. Scale bar for 15kx images indicates 1μm, for 80kx images scale bar is 200nm.

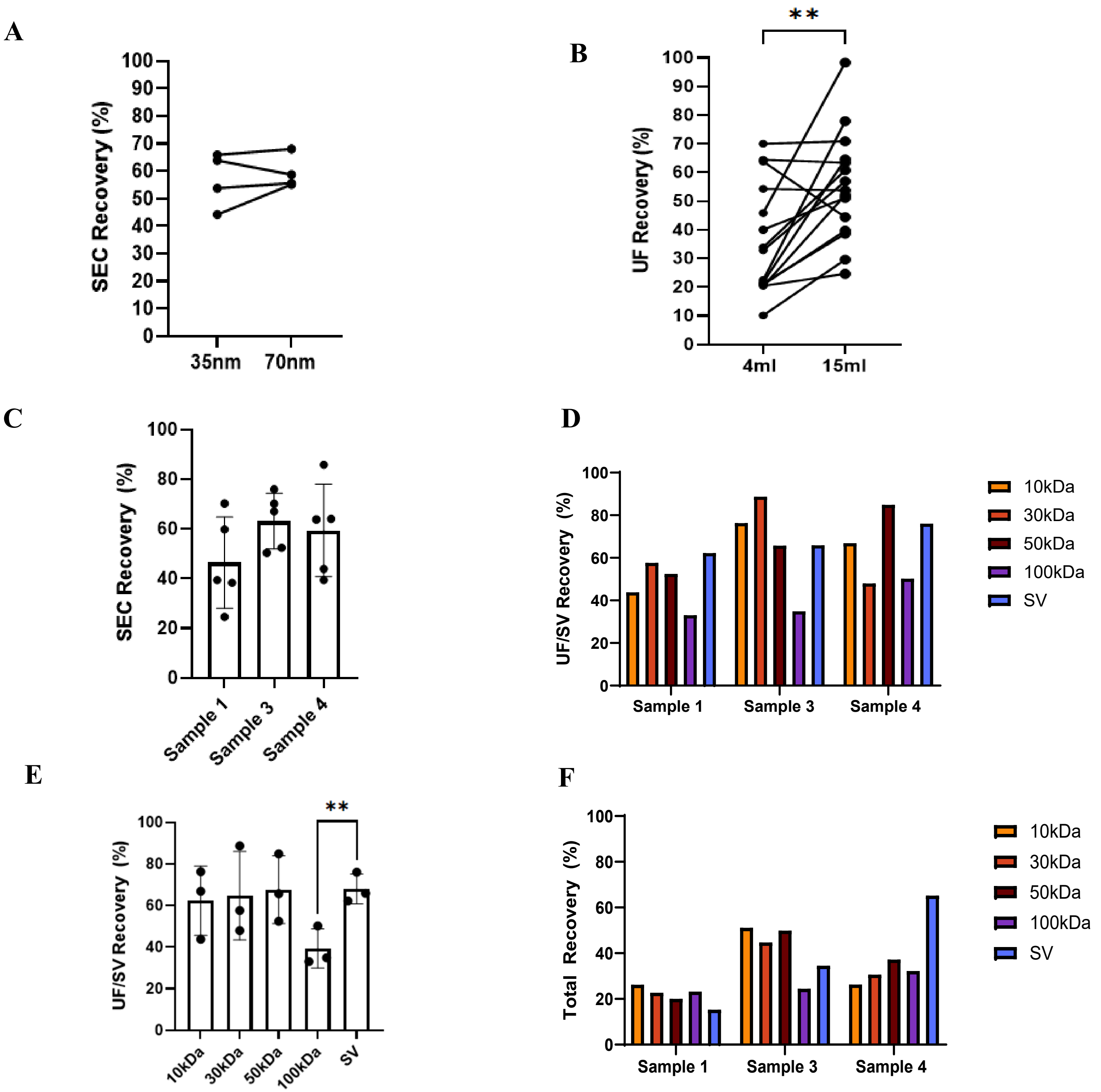

Supplementary Figure 2. nFCM and MRPS- based comparative analysis of concentration methods for particle recovery from three cancer-free human stool specimens (a) Particle recovery for 35nm and 70nm SEC columns as measured by nFCM, paired t test (b) Recovery for 4ml vs 15ml centrifugal filters as measured by nFCM, paired t test, $**p < 0.001$ (c) SEC recovery as measured by MRPS, Mean ± SD (d) Post-SEC recovery for each patient and concentration method as measured by MRPS (e) Post-SEC recovery among the different concentration methods as measured by MRPS, Mean ± SD, One Way ANOVA, $**p < 0.001$ (f) Total recovery rates for each patient and concentration method, as measured by MRPS.

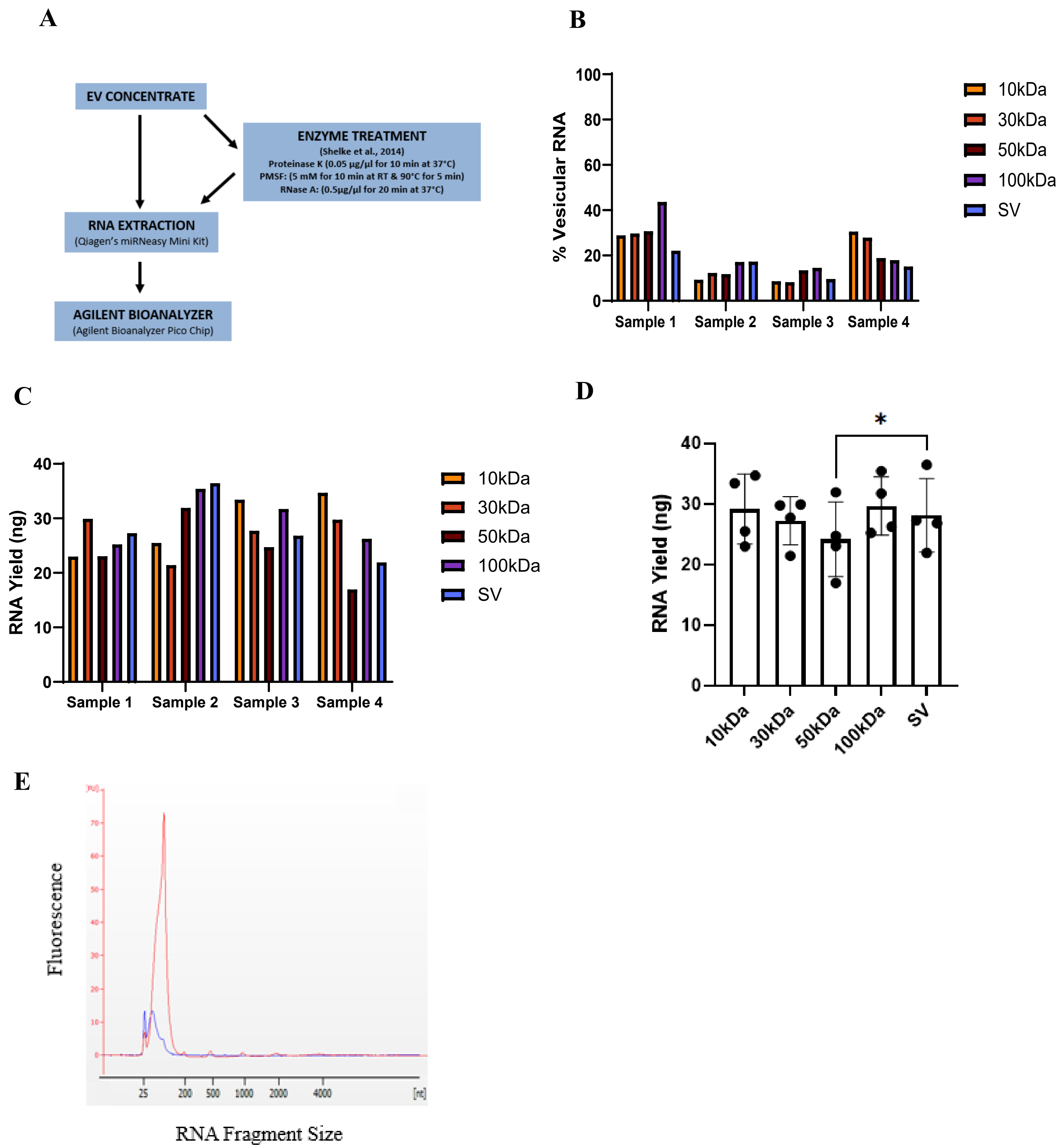

Supplementary Figure 3. Characterization of RNA content from stool-derived EVs (a) Schematic workflow for determining the extent of non-vesicular RNA contamination for each concentration method (b) Percent vesicular RNA for each patient and concentration method (c) Vesicular RNA yield for each patient and concentration method among the different EV concentration methods (d) RNA yield for each concentration method across patient samples, One Way ANOVA, $*p < 0.05$ (e) Electropherogram depicting RNA fragment size profile pre-enzymatic (red) and post- enzymatic (blue) treatment measured by fluorescence on the Agilent Bioanalyzer Pico chip.

A

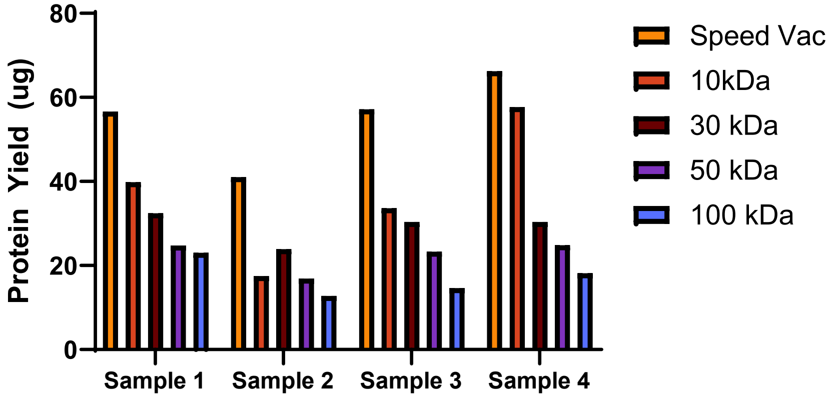

B

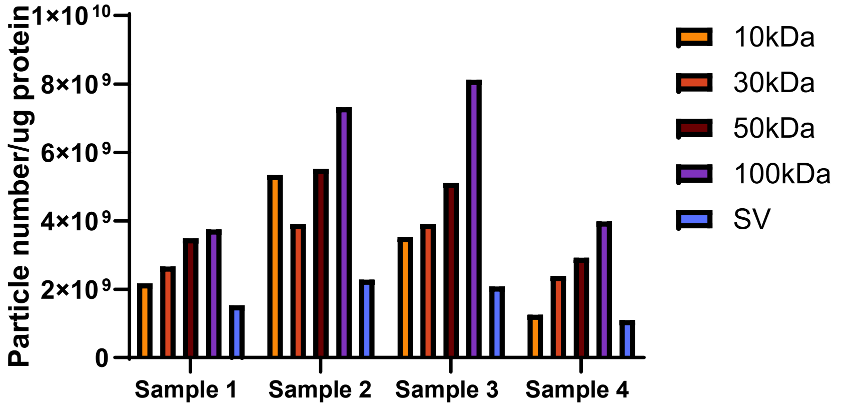

Supplementary Figure 4. Characterization of protein yield and contamination from stool-derived EVs (a) Protein yield for each patient and concentration method (b) Ratio of total particle number and total protein yield for each patient and concentration method, a higher ratio suggests less co-isolated soluble protein contamination.

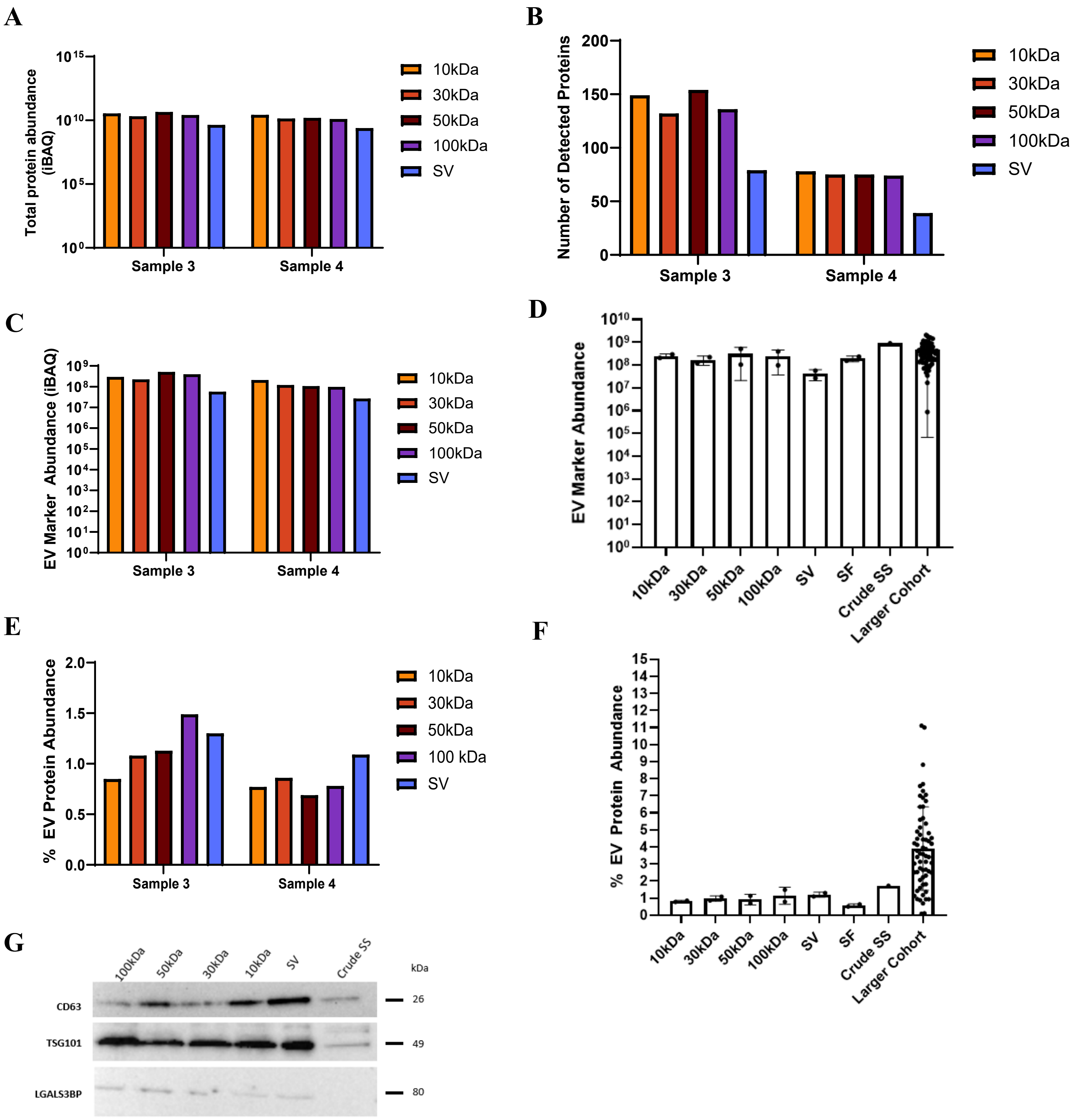

Supplementary Figure 5. Stool EV proteomic landscape (a) Total protein abundance (iBAQ values) for each patient and concentration method submitted for mass spectrometry (b) Number of proteins detected in for each sample and concentration method (c) Total EV marker protein abundance (iBAQ values) for each patient and concentration method submitted for mass spectrometry (d) Total EV marker protein abundance (iBAQ values) across concentration methods and larger cohort, Mean ± SD (e) Percent EV marker protein abundance across each patient and concentration method (f) Percent protein abundance of previously identified EV markers across concentration methods and larger cohort, Mean ± SD (g) Western blot (15ug) showing human EV protein expression post-isolation for the five concentration methods and pre-isolation (stool supernatant) that were not detected by mass spectrometry.

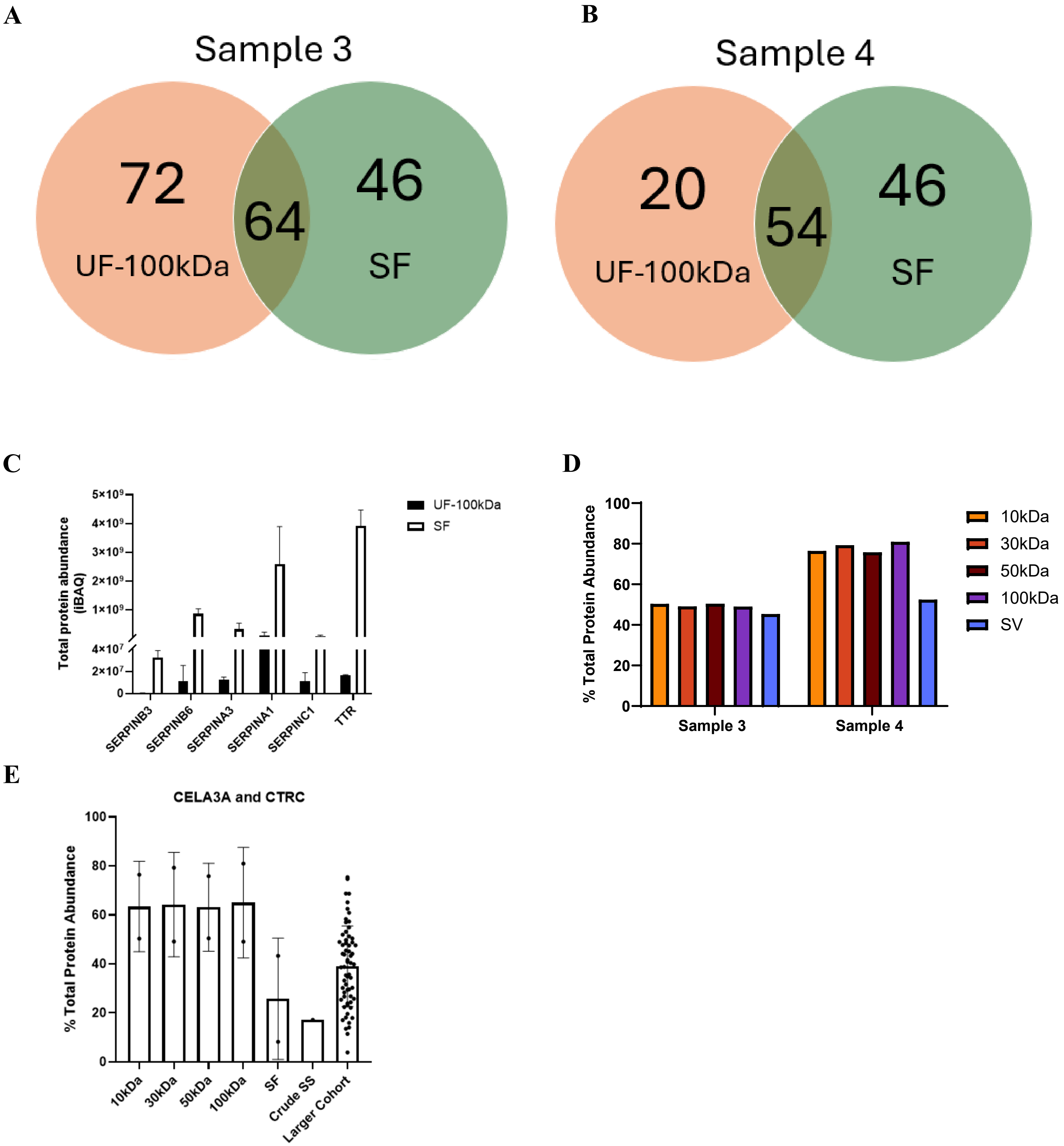

Supplementary Figure 6. Stool EV purity evaluation, UF-100kDa versus SF (a) Patient 3 venn diagram comparison of UF-100kDa and soluble fraction (b) Patient 4 venn diagram comparison of UF-100kDa and soluble fraction (c) Protein abundance (iBAQ) of potential purity markers for stool EV preparations (d) Percent protein abundance of CTRC and CELA3A across each patient and concentration method (e) Percent abundance of the top pancreatic hydrolases (CELA3A, CTRC) in stool EV samples, Mean ± SD.

A

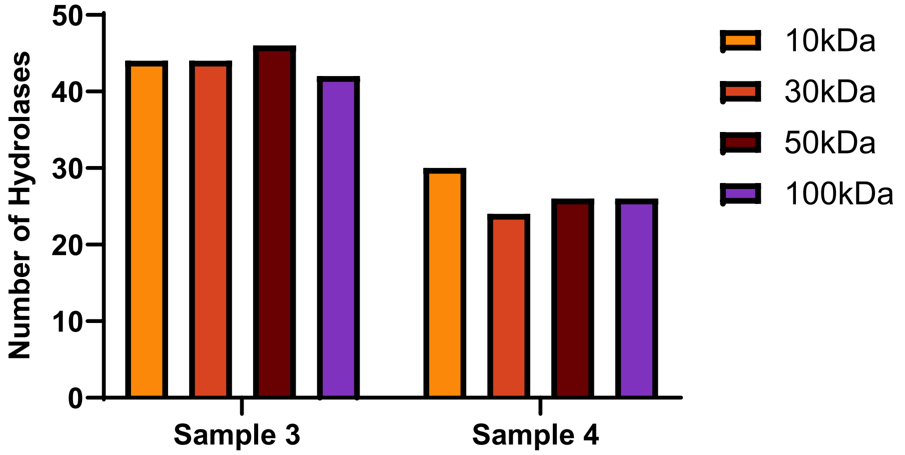

B

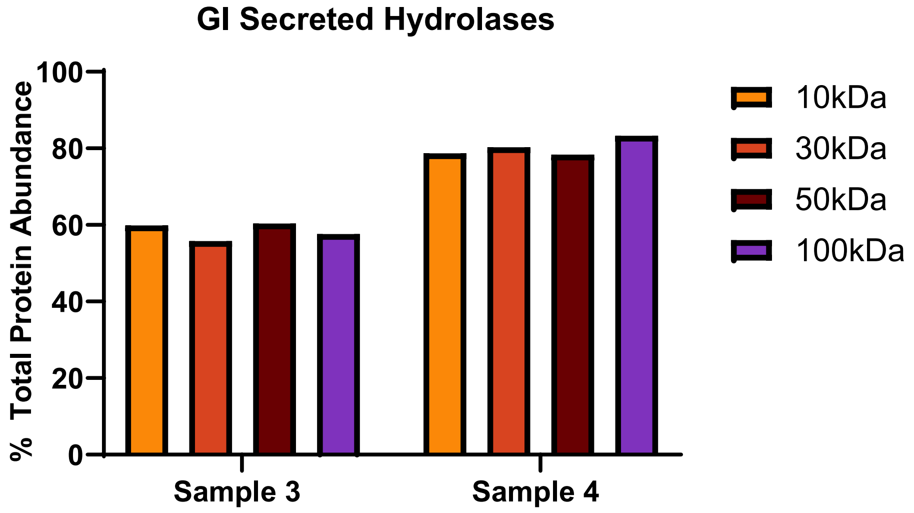

Supplementary Figure 7. Hydrolase detection in stool EV preparations (a) Number of hydrolases detected among ultrafiltration cutoffs in each patient (b) Percent protein abundance of GI secreted hydrolases detected among ultrafiltration cutoffs in each patient.

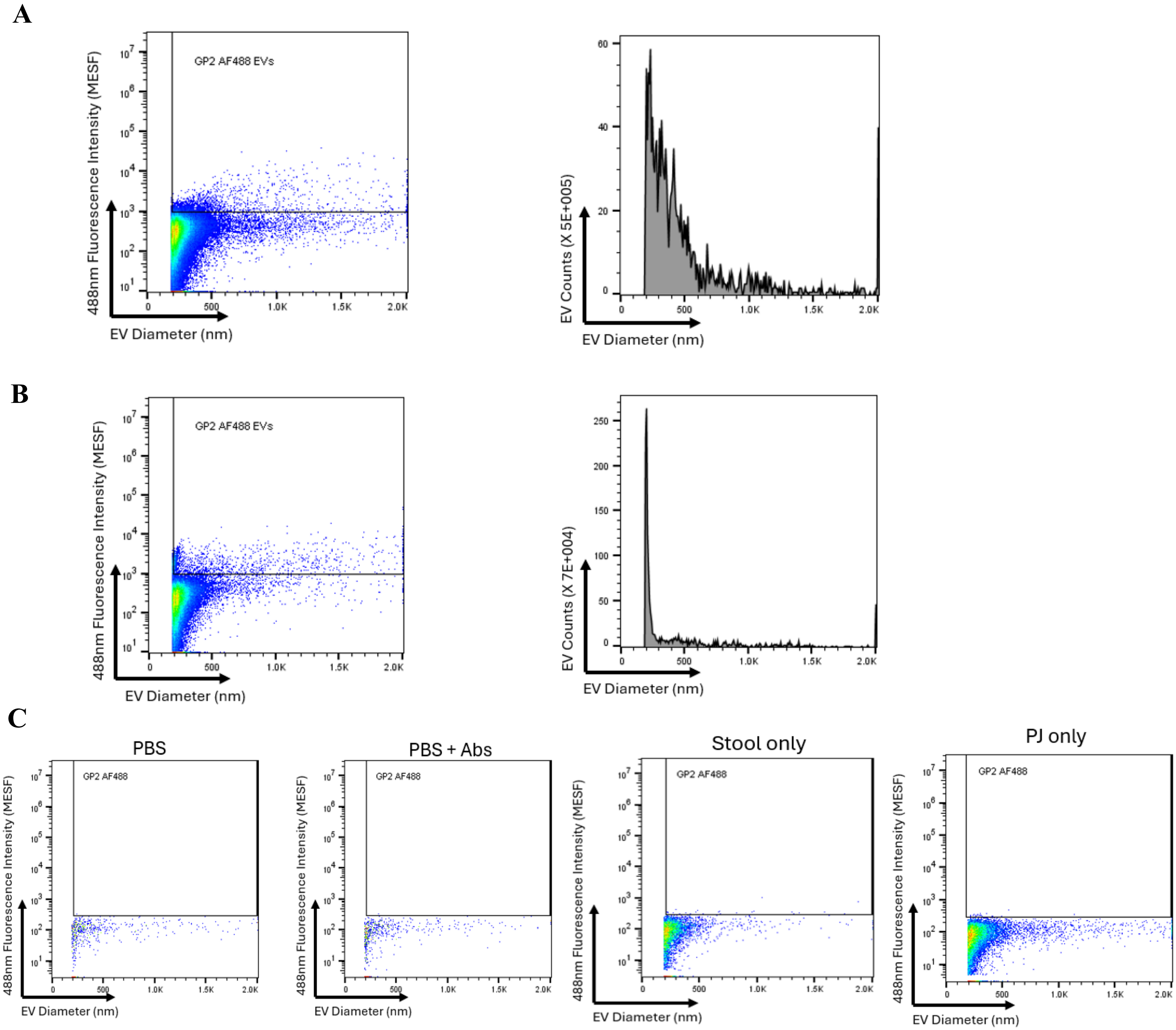

Supplementary Figure 8. GP2 detection on a per vesicle level using nanoscale flow cytometry (a) GP2 detection scatterplot in a representative stool sample incubated with antibody and histogram showing the relationship between EV count and EV diameter (b) GP2 detection scatterplot in a representative pancreatic juice (PJ) sample incubated with antibody and histogram showing the relationship between EV count and diameter (c) GP2 scatterplot for controls: PBS only sample, PBS and antibody sample, stool sample only, and PJ sample only. Non-stained samples determined the LOD of gating and we applied gating accordingly to stained samples.

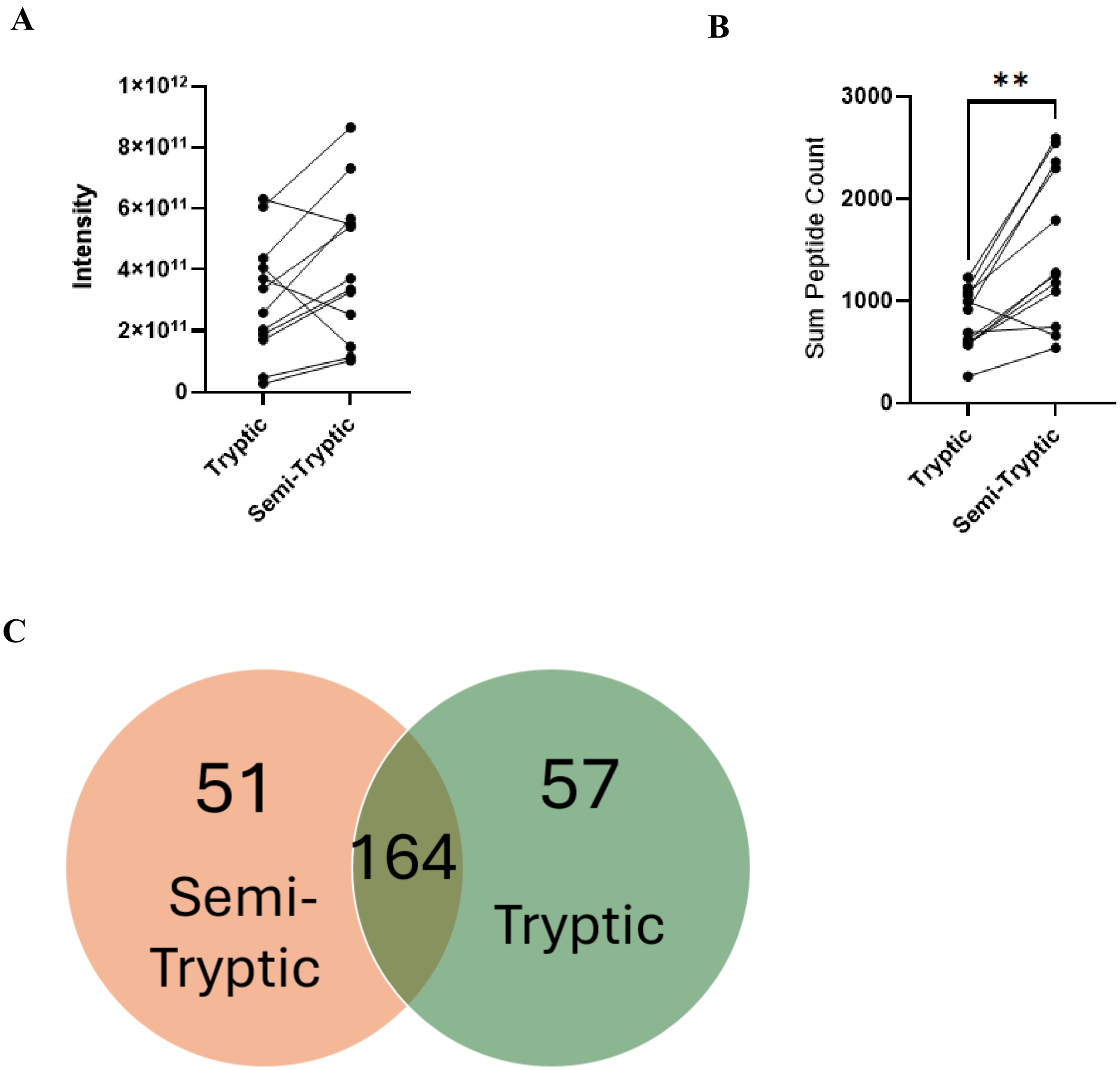

Supplementary Figure 9. Semi-tryptic and tryptic data analyses for concentration method comparison cohort (a) Intensities for the different analysis approaches, Paired t test (b) Sum peptide counts for the different analysis approaches, Paired t test, $^{**}P < 0.01$ (c) Venn diagram showing unique and similar human proteins from EV samples among the data analyses.

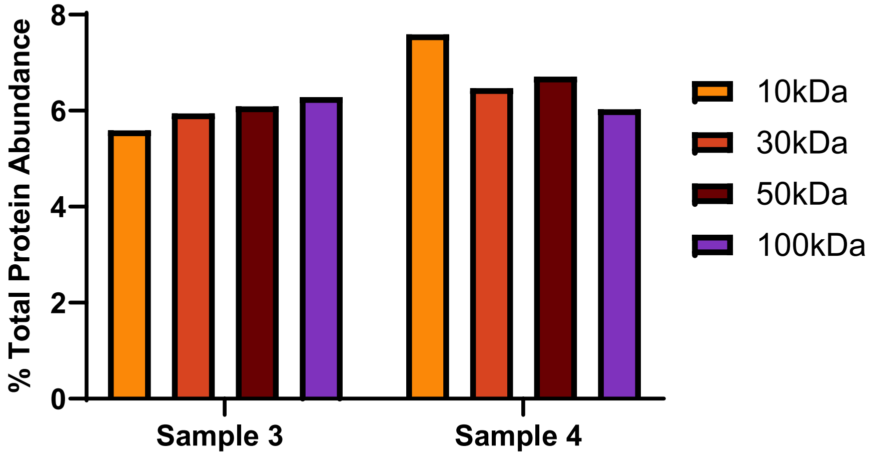

Supplementary Figure 10. Percent of total protein abundance associated with colon enriched/specific proteins among ultrafiltration cutoffs in each patient.

A

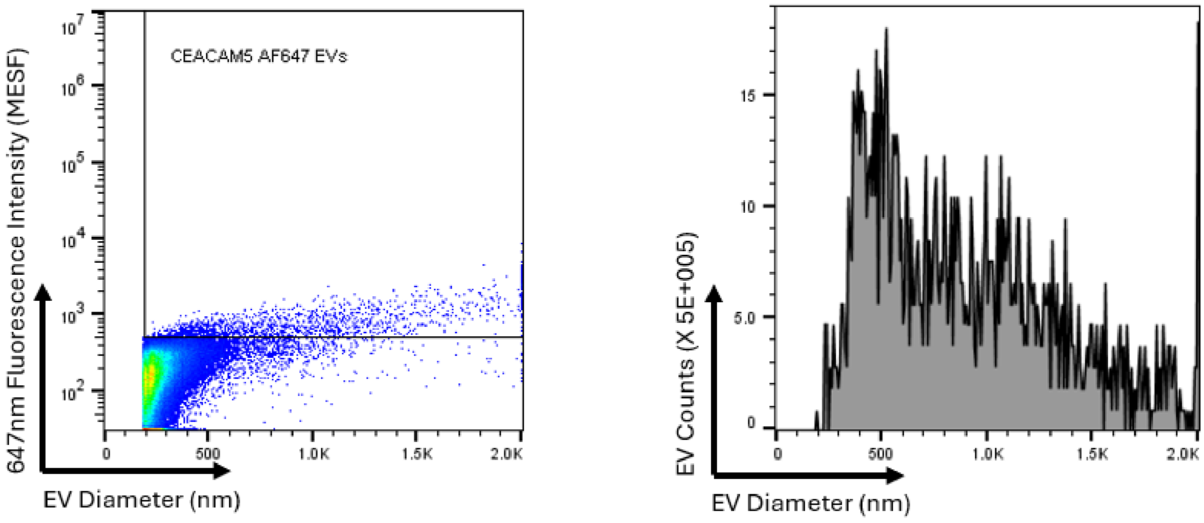

B

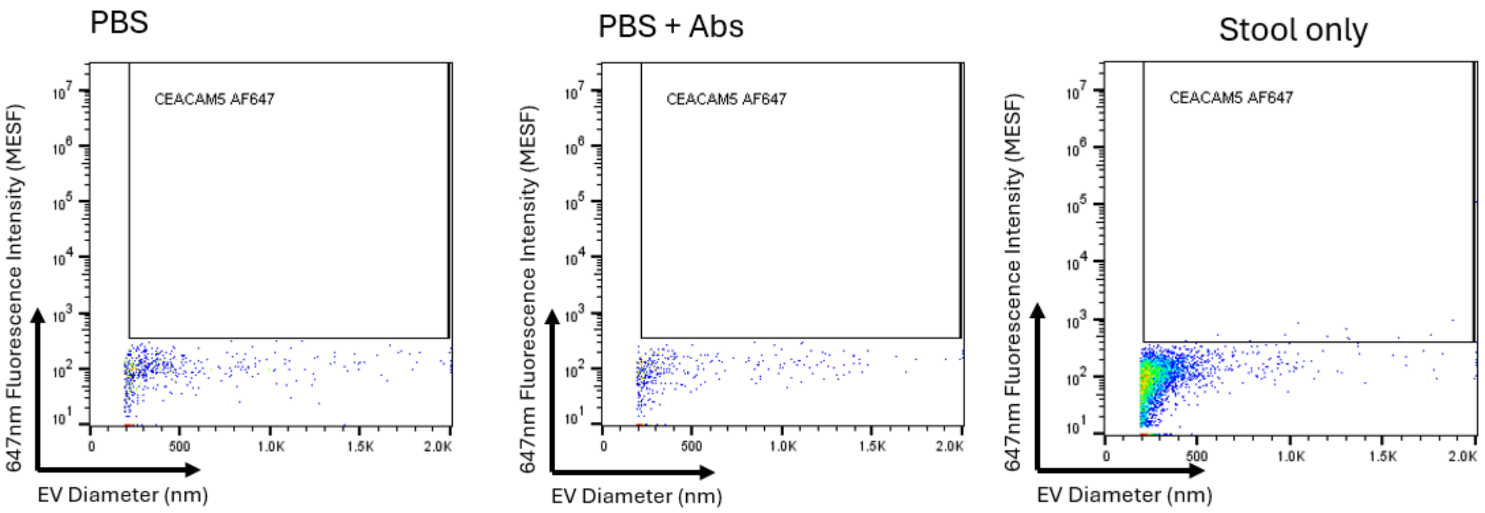

Supplementary Figure 11. CEACAM5 detection on a per vesicle level using nanoscale flow cytometry (a) CEACAM5 detection scatterplot in a representative stool sample incubated with antibody and histogram showing the relationship between EV count and EV diameter (b) CEACAM5 scatterplot for controls: PBS only sample, PBS and antibody sample, and stool sample only. Non-stained samples determined the LOD of gating and we applied gating accordingly to stained samples.

A

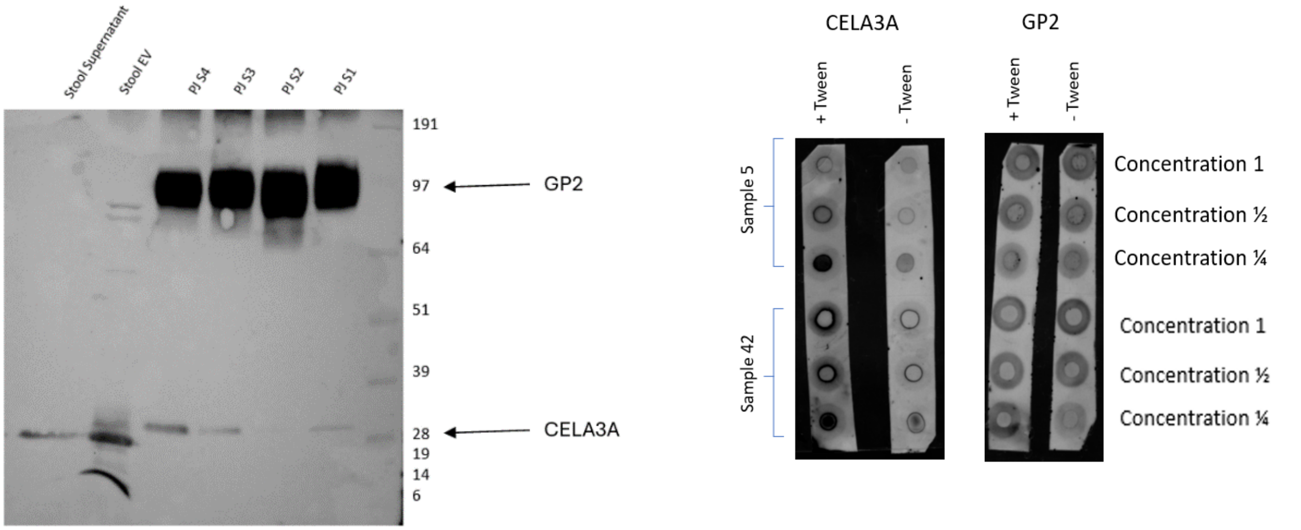

B

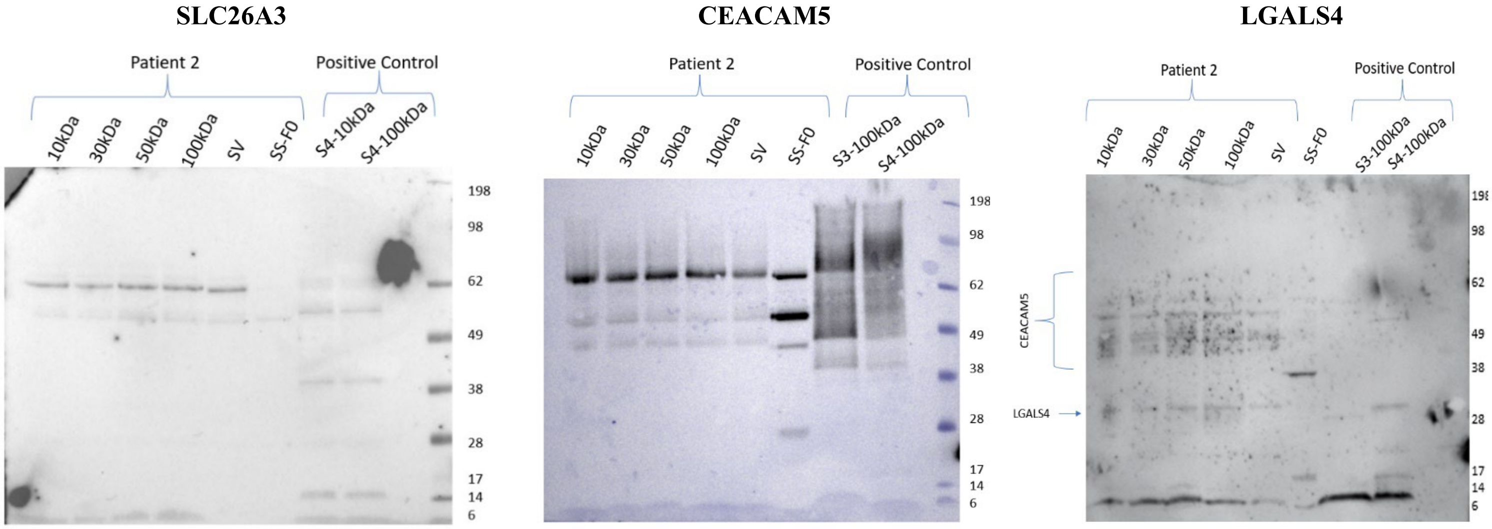

C

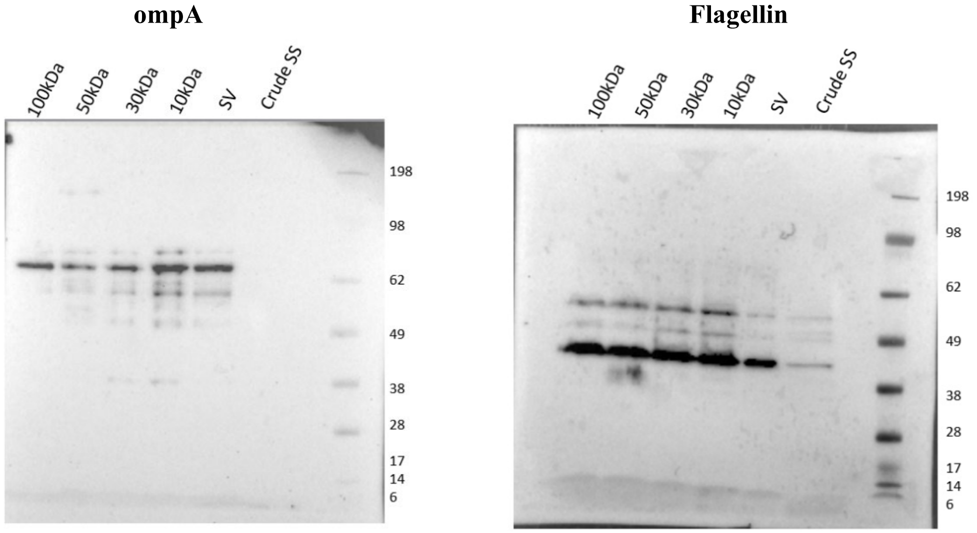

Supplementary Figure 12. Uncropped western blot/dot blot images (a) Pancreatic proteins: CELA3A and GP2 (b) Colon proteins: SLC26A3, CEACAM5, LGALS4 (c) Conserved bacterial proteins: ompA, Flagellin

CD63

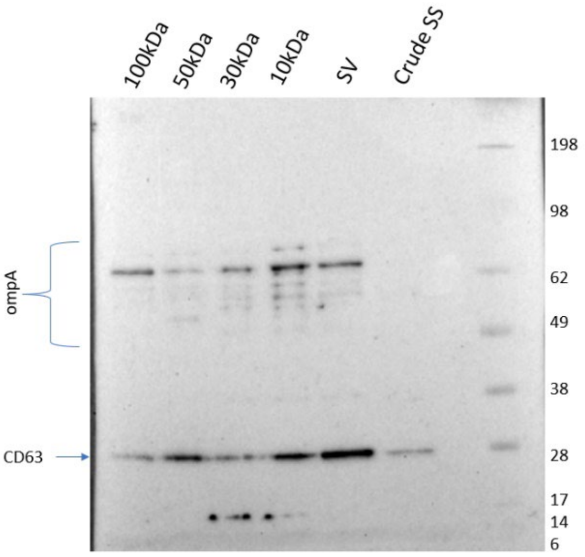

TSG101

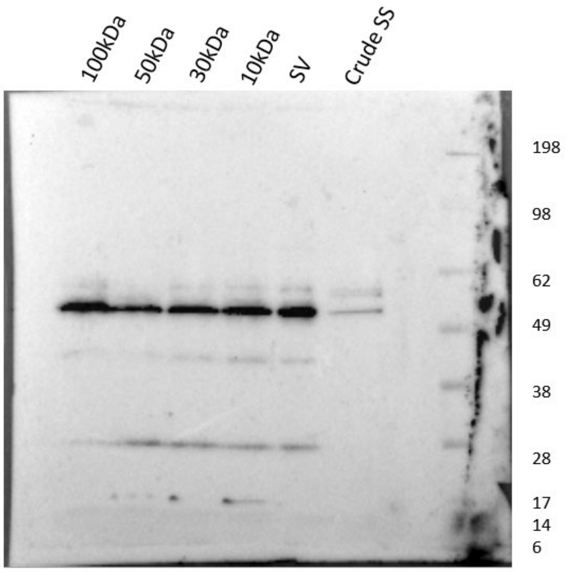

LGALS3BP

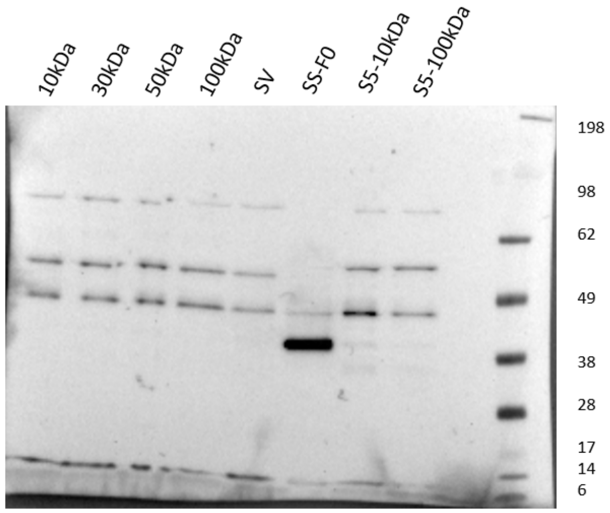

Supplementary Figure 13. Uncropped western blot images for EV markers: CD63, TSG101, LGALS3BP
