# Supplementary material for: The proteomic landscape of stool-derived extracellular vesicles in patients with pre-cancerous lesions and colorectal cancer
Source: Commun Biol. 2025 Feb 13;8:228. doi: 10.1038/s42003-025-07652-5 (PMC11825688; doi:10.1038/s42003-025-07652-5)
Supplement: Supplementary file 2 — Description of Additional Supplementary Files [file 42003_2025_7652_MOESM2_ESM.pdf]

Description of Additional Supplementary Files

File name: Supplementary Data

Description: The source data behind the figures in the paper.

File name: Supplementary Data 1

Description: Proteins identified among stool samples submitted for concentration method comparison experiment

File name: Supplementary Data 2

Description: Stool EV proteins identified in larger cohort of cancer-free, precancerous lesion, and cancer patients

File name: Supplementary Data 3

Description: Stool proteins identified in semi-tryptic and tryptic datasets in concentration method comparison experiment

File name: Supplementary Data 4

Description: Bacterial stool proteins identified in concentration method comparison experiment
