# Supplementary material for: The proteomic landscape of stool-derived extracellular vesicles in patients with pre-cancerous lesions and colorectal cancer
Source: Commun Biol. 2025 Feb 13;8:228. doi: 10.1038/s42003-025-07652-5 (PMC11825688; doi:10.1038/s42003-025-07652-5)
Supplement: Supplementary file 8 — Reporting summary [file 42003_2025_7652_MOESM8_ESM.pdf]

nature portfolio

Corresponding author(s): Emmalee Northrop-Albrecht

Last updated by author(s): Jan 10, 2025

Reporting Summary

Nature Portfolio wishes to improve the reproducibility of the work that we publish. This form provides structure for consistency and transparency in reporting. For further information on Nature Portfolio policies, see our Editorial Policies and the Editorial Policy Checklist.

Statistics

For all statistical analyses, confirm that the following items are present in the figure legend, table legend, main text, or Methods section.

n/a	Confirmed	
☐	☒	The exact sample size (*n*) for each experimental group/condition, given as a discrete number and unit of measurement
☐	☒	A statement on whether measurements were taken from distinct samples or whether the same sample was measured repeatedly
☐	☒	The statistical test(s) used AND whether they are one- or two-sided<br>*Only common tests should be described solely by name; describe more complex techniques in the Methods section.*
☒	☐	A description of all covariates tested
☒	☐	A description of any assumptions or corrections, such as tests of normality and adjustment for multiple comparisons
☐	☒	A full description of the statistical parameters including central tendency (e.g. means) or other basic estimates (e.g. regression coefficient) AND variation (e.g. standard deviation) or associated estimates of uncertainty (e.g. confidence intervals)
☐	☒	For null hypothesis testing, the test statistic (e.g. *F*, *t*, *r*) with confidence intervals, effect sizes, degrees of freedom and *P* value noted<br>*Give P values as exact values whenever suitable.*
☒	☐	For Bayesian analysis, information on the choice of priors and Markov chain Monte Carlo settings
☒	☐	For hierarchical and complex designs, identification of the appropriate level for tests and full reporting of outcomes
☒	☐	Estimates of effect sizes (e.g. Cohen's *d*, Pearson's *r*), indicating how they were calculated

Our web collection on statistics for biologists contains articles on many of the points above.

Software and code

Policy information about availability of computer code

Data collection	No software was used for data collection.
Data analysis	GraphPad Prism 9.5.1 (Carlsbad, CA) was used for statistical analyses and figure generation.

For manuscripts utilizing custom algorithms or software that are central to the research but not yet described in published literature, software must be made available to editors and reviewers. We strongly encourage code deposition in a community repository (e.g. GitHub). See the Nature Portfolio guidelines for submitting code & software for further information.

Data

Policy information about availability of data

All manuscripts must include a data availability statement. This statement should provide the following information, where applicable:

- Accession codes, unique identifiers, or web links for publicly available datasets
- A description of any restrictions on data availability
- For clinical datasets or third party data, please ensure that the statement adheres to our policy

The authors declare that most of the main data supporting the conclusions of the current study is available within the manuscript and its supplementary files. The numerical source data for graphs and charts is shown in Supplementary Data. The uncropped western blot and dot blot results are shown as Supplementary Figs 12 and 13. This study includes no data deposited in external repositories. For access to other data contact the corresponding author. It may be made available, but it is conditional to approval from the Mayo Clinic Institutional Review Board and will require a data use agreement from Mayo Clinic Legal Contracts Administration.

Human research participants

Policy information about studies involving human research participants and Sex and Gender in Research.

Reporting on sex and gender	Sex was collected on all patient samples, but no sex-based or gender based analyses were performed.
Population characteristics	No covariates were used in the analyses of this study.
Recruitment	Patients were recruited based on clinical appointments and diagnosis and if they meet eligibility requirements. Other than targeting specific patients based on their diagnosis, there were no biases.
Ethics oversight	This work was approved by the Mayo Clinic Institutional Review Board with informed patient consent under IRB 18-008752 and IRB 18-011965.

Note that full information on the approval of the study protocol must also be provided in the manuscript.

Field-specific reporting

Please select the one below that is the best fit for your research. If you are not sure, read the appropriate sections before making your selection.

☒ Life sciences ☐ Behavioural & social sciences ☐ Ecological, evolutionary & environmental sciences

For a reference copy of the document with all sections, see nature.com/documents/nr-reporting-summary-flat.pdf

Life sciences study design

All studies must disclose on these points even when the disclosure is negative.

Sample size	For the larger mass spectrometry cohort, sample size calculations were based on the assumption that iBAQ scores follow a log-normal distribution with mean 0 and a range of standard deviations of 0.47, 0.67, and 0.83 under the null hypothesis. These standard deviations on the log-scale are roughly equivalent to coefficient of variation of the iBAQ scores on the raw scale of 50%, 75%, and 100%. The minimum requirement for this experiment was to have 80% power to detect a FC between any two patient groups of 2 or higher using a 2-sided significant level of 5%. To achieve this, sample sizes of 9, 16, and 24 per patient group were needed with an average sample size of 16. No corrections were made to account for false-discovery as any identified in this experiment will undergo biological validation using independent patient samples.
Data exclusions	There were no data exclusions.
Replication	We previously confirmed that stool EV isolation was reproducible (Northrop-Albrecht et al., 2022).
Randomization	Randomization of samples was performed using the Microsoft excel function.
Blinding	For smaller concentration method cohort, cancer-fee individuals were used because cancer status was irrelevant. Lab personnel were blinded for larger mass spectrometry discovery cohort.

Reporting for specific materials, systems and methods

We require information from authors about some types of materials, experimental systems and methods used in many studies. Here, indicate whether each material, system or method listed is relevant to your study. If you are not sure if a list item applies to your research, read the appropriate section before selecting a response.

Materials & experimental systems

n/a	Involved in the study	
☐	☒	Antibodies
☒	☐	Eukaryotic cell lines
☒	☐	Palaeontology and archaeology
☒	☐	Animals and other organisms
☒	☐	Clinical data
☒	☐	Dual use research of concern

Methods

n/a	Involved in the study	
☒	☐	ChIP-seq
☒	☐	Flow cytometry
☒	☐	MRI-based neuroimaging

Antibodies

Antibodies used	Antibodies and their dilutions are listed in the methods section of the manuscript.
Validation	Validation of antibodies was provided by commercial vendors using knockout gene systems or recombinant proteins.
